# Cdc7p-Dbf4p Regulates Mitotic Exit by Inhibiting Polo Kinase

**DOI:** 10.1371/journal.pgen.1000498

**Published:** 2009-05-29

**Authors:** Charles T. Miller, Carrie Gabrielse, Ying-Chou Chen, Michael Weinreich

**Affiliations:** 1 Graduate Program in Cell and Molecular Biology, Michigan State University, East Lansing, Michigan, United States of America; 2 Laboratory of Chromosome Replication, Van Andel Research Institute, Grand Rapids, Michigan, United States of America; 3 Graduate Program in Genetics, Michigan State University, East Lansing, Michigan, United States of America; National Institute of Diabetes and Digestive and Kidney Diseases, United States of America

## Abstract

Cdc7p-Dbf4p is a conserved protein kinase required for the initiation of DNA replication. The Dbf4p regulatory subunit binds Cdc7p and is essential for Cdc7p kinase activation, however, the N-terminal third of Dbf4p is dispensable for its essential replication activities. Here, we define a short N-terminal Dbf4p region that targets Cdc7p-Dbf4p kinase to Cdc5p, the single Polo kinase in budding yeast that regulates mitotic progression and cytokinesis. Dbf4p mediates an interaction with the Polo substrate-binding domain to inhibit its essential role during mitosis. Although Dbf4p does not inhibit Polo kinase activity, it nonetheless inhibits Polo-mediated activation of the mitotic exit network (MEN), presumably by altering Polo substrate targeting. In addition, although *dbf4* mutants defective for interaction with Polo transit S-phase normally, they aberrantly segregate chromosomes following nuclear misorientation. Therefore, Cdc7p-Dbf4p prevents inappropriate exit from mitosis by inhibiting Polo kinase and functions in the spindle position checkpoint.

## Author Summary

Cdc7p-Dbf4p is a two-subunit enzyme required to copy the genetic material present on every chromosome in a process termed DNA replication. Dbf4p is an essential regulatory subunit of this enzyme that likely directs the Cdc7p subunit to its targets within the cell. We found that Dbf4p physically interacts with another protein called Polo that acts during mitosis, a later step in the cell cycle when the newly copied chromosomes are equally divided to mother and daughter cells. Polo is a master regulator of mitosis and impacts many other proteins required for cell division. We determined that Cdc7p-Dbf4p is a Polo inhibitor and, further, that Cdc7p-Dbf4p delayed or prevented chromosome segregation when errors occurred during the cell division process. Interestingly, Dbf4p may bind the Polo substrate-binding domain using a type of interaction not previously described. Thus, we have uncovered a new activity for Cdc7p-Dbf4p in the cell cycle to inhibit chromosome segregation, and these findings impact multiple fields that investigate how cells accurately copy and segregate their chromosomes.

## Introduction

Accurate ordering of cell cycle events is an important requirement for the viability of all eukaryotic organisms. Once cells commit to duplicate their genome they must restrain mitosis until replication is complete and then accurately coordinate mitosis with cytokinesis to ensure the faithful transmission of chromosomes to daughter cells [1]. Importantly, errors in cell cycle checkpoints that enforce this ordering can be deleterious for accurate chromosome transmission. For instance, DNA damage or replication fork arrest during S-phase elicits a reversible block to mitotic progression by the budding yeast Mec1p (HsATR) and Rad53p (HsChk2) checkpoint kinases [2],[3]. In the absence of Mec1p or Rad53p, replication fork arrest during S-phase is not sensed leading to premature mitotic events and cell death (reviewed by [4]). Additionally, since daughter cell growth is highly polarized in the budding yeast, exit from mitosis is prevented until sister chromatids segregate through the bud neck and into the daughter cell [5]–[7]. This ensures that spindle disassembly and mitotic exit are not initiated until accurate chromosome partitioning between mother and daughter cells has occurred. Failure to block mitotic exit when nuclear division takes place within the mother cell results in polyploid and anucleate progeny [8],[9]. It is not surprising therefore, that both entry into and exit from mitosis are delayed by cellular checkpoints that respond to replication stress, chromosome damage, or spindle disruption [1]. Errors in these mitotic checkpoints are catastrophic and result in ploidy defects and genetic alterations, which are frequently observed in human cancers (reviewed by [10]).

The Cdc7p-Dbf4p kinase is required to catalyze the initiation of DNA synthesis at the beginning of S-phase (reviewed by [11]). Cdc7p kinase activity is tightly regulated during the cell cycle by binding the Dbf4p regulatory subunit, which is cyclically expressed. Dbf4p accumulates in late G1, is present throughout S-phase and then is destroyed during mitosis and early G1 by anaphase promoting complex (APC)-dependent degradation [12]–[17]. Therefore, Cdc7p-Dbf4p kinase activity is low following exit from mitosis and entry into G1-phase until it is needed to initiate a new round of DNA synthesis in late G1-phase of the following cell cycle. Multiple lines of evidence suggest that Cdc7p-Dbf4p activates the MCM DNA helicase [18]–[20] that is assembled at origins of replication in early G1 in an inactive form (reviewed in [21],[22]).

In addition to its essential role in replication initiation, several studies suggest that the Cdc7p-Dbf4p kinase responds to DNA damage or replication fork stalling but its precise role in these activities is unknown [17], [23]–[25]. Dbf4p encodes a dispensable BRCT-like domain in the N-terminus that might target the kinase to stalled replication forks [26],[27]. In fission yeast, the Cdc7p-Dbf4p ortholog Hsk1p-Dfp1p interacts with Swi1p (budding yeast Tof1p), a component of replication forks required for fork stability and also promotes centromeric cohesion in early mitosis [28],[29]. Rad53p also phosphorylates Dbf4p in response to replication stress and this regulation requires N-terminal Dbf4p sequences through which Rad53p physically interacts [17],[25],[30]. Interestingly, the absence of the BRCT-like domain results in a defect in late origin activation suggesting that this domain might alter Cdc7p-Dbf4p binding at early versus late replication origins [26]. Together, these data suggest that the Dbf4p N-terminus encodes non-essential regulatory functions that target the kinase to particular substrates.

To identify proteins that interact with the Dbf4p N-terminus, we performed a yeast two-hybrid screen with an N-terminal region of Dbf4p and identified an interaction with the Cdc5p kinase, the only Polo ortholog in yeast. Budding yeast Polo, like *Drosophila* Polo and human Polo-like kinase 1 (Plk1), functions as a master regulator of mitotic progression and is also required for cytokinesis (reviewed by [31],[32]). Polo activity is regulated by several independent cellular mechanisms. Polo protein levels are controlled by APC-dependent degradation in mitosis/G1-phase and activation of Polo catalytic activity requires phosphorylation by Cdk1 kinase early in G2 [33]–[35]. In addition, Polo function is inhibited by cell cycle checkpoints that are induced following DNA or spindle damage [36]–[38]. A genetic and physical interaction between Dbf4 and Polo was described previously [39],[40], however the biological significance of this interaction was not known.

Polo controls multiple mitotic events to ensure accurate chromosome segregation. After anaphase initiation, Polo is required to activate the FEAR (Cdc14 early anaphase release) and MEN (mitotic exit network) pathways that promote nucleolar release of Cdc14p phosphatase [37], [41]–[43]. Limited Cdc14p release by the FEAR pathway promotes accurate rDNA and telomere segregation [44]–[46]. Subsequent full nucleolar release of Cdc14p by the MEN reverses Cdk substrate phosphorylation that leads to APC-Cdh1p activation, cyclin destruction and mitotic spindle disassembly (reviewed by [47]). Activation of the MEN is promoted by Tem1p-GTP and antagonized by Bfa1p-Bub2p, a two-component GTPase activating protein (GAP) [48]–[50]. To promote mitotic exit, Polo phosphorylates Bfa1p-Bub2p to inhibit its GAP activity and is also required for activation of Dbf2p kinase activity, independently of Bfa1p-Bub2p [37],[49],[51],[52]. The Polo requirement for Dbf2p kinase activation may reflect that Polo also promotes Cdc14p release in the FEAR pathway, which primes the MEN [43]. Therefore, Polo promotes accumulation of Tem1p-GTP and activation of the downstream MEN kinases Cdc15p and Dbf2p, which ultimately cause full release of Cdc14p from the nucleolus. In response to replication fork arrest, Rad53 inhibits MEN activation, which may or may not impact Polo activity since the molecular basis of this regulation is not understood [53],[54]. Spindle position defects also counteract Polo activity by targeting Kin4p kinase to the spindle poles where it inhibits Polo-dependent Bfa1p phosphorylation [8],[9],[55]. Failure to execute the spindle position checkpoint (SPOC) results in premature exit from mitosis and nuclear partitioning defects.

Here we define an N-terminal Dbf4p polo-box interaction region (that we refer to as the “PIR”) that binds directly to Polo and show that Dbf4p inhibits Polo and Dbf2p activity. Deletion of the PIR allows Cdc14p nucleolar release in a *cdc5-1* mutant at the non-permissive temperature. In response to nuclear mispositioning, a *dbf4* mutant lacking the PIR fails to arrest in mitosis and prematurely exits the cell cycle. Thus, Dbf4 protein is required for proper functioning of the spindle position checkpoint most likely by antagonizing the ability of Polo to promote Cdc14p release in either the FEAR or MEN pathways. Our work therefore reveals a previously unrecognized function for Dbf4p in the regulation of mitotic progression through a direct interaction with Polo.

## Results

### The Cdc5p Polo-Box Domain Interacts with the Dbf4p N-Terminus

**Figure 1:**
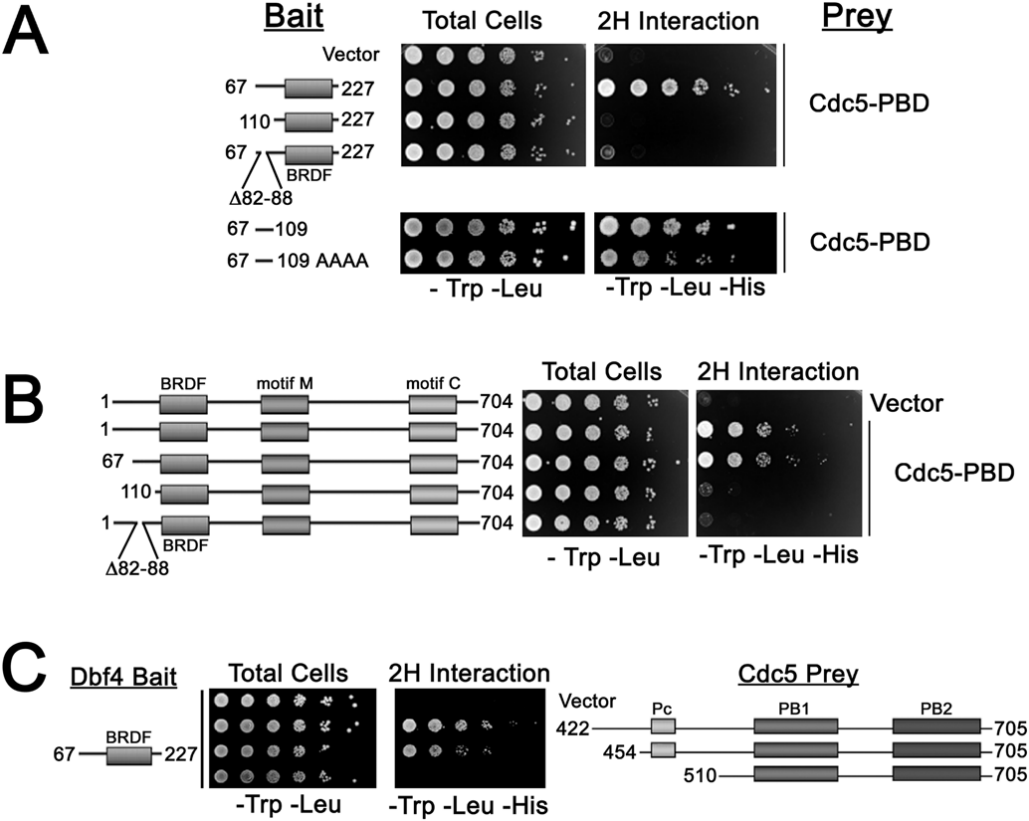
The N-terminus of Dbf4p interacts with the Polo PBD. Cells co-transformed with various bait *GAL4* DNA binding domain fusions (GBD-*DBF4*) and prey *GAL4* activation domain fusions (GAD-*CDC5*) constructs were spotted at 10-fold serial dilutions on SCM–Trp –Leu and SCM–Trp–Leu–His plates containing 2 mM 3AT (A, C) or 0.5 mM 3AT (B) and incubated at 30°C for 2 to 3 days. The GBD-Dbf4 protein levels are shown in Figure S1.

We conducted a yeast two-hybrid screen to identify proteins that interact with the Dbf4p N-terminus (residues 67–227) and recovered multiple clones encoding the polo-box domain (PBD) of Cdc5p. Polo kinase has two conserved domains; an N-terminal kinase domain and a C-terminal region called the polo-box domain (PBD) (reviewed by [56]), which is a phospho-Ser/Thr binding module that targets the kinase to its mitotic substrates [57],[58]. The crystallographic structure of the Plk1 PBD bound to a phospho-threonine peptide has been solved [59]. Since the Dbf4p BRCT-like region alone (residues 110–227) failed to interact with the Polo PBD (Figure 1A), this suggested that the PBD interaction was occurring through Dbf4p N-terminal sequences from 67–109. Residues 67–109 were similarly required for the Polo interaction within the context of full length Dbf4p (Figure 1B) and were sufficient to interact with the Polo PBD (Figure 1A). Dbf4p residues 67–109 with all serines/threonines changed to alanine still interacted with the PBD (Figure 1A) suggesting that the PBD can bind to this Dbf4p region independently of phosphorylation. Further deletion and point mutant analysis (Y-C.C. and M.W., unpublished data) revealed that residues 82–88 are essential for the Dbf4p-Polo interaction (Figure 1A, B).

The PBD is composed of three conserved regions called the Polo-cap (Pc), Polo-box 1 (PB1) and Polo-box 2 (PB2) that fold together to form a functional phosphopeptide-binding domain [59]. We deleted conserved residues within the PBD to test their requirement for interaction with Dbf4p (Figure 1C). Deletion of residues preceding the PBD (GAD-Polo_454–705_) had little effect on the Dbf4p-Polo interaction. However, elimination of the Pc (GAD-Polo_510–705_) completely disrupted the interaction with Dbf4p. These data suggest that the structural integrity of the Polo PBD is required for Dbf4p binding.

### Dbf4p Directly Interacts with Polo

**Figure 2:**
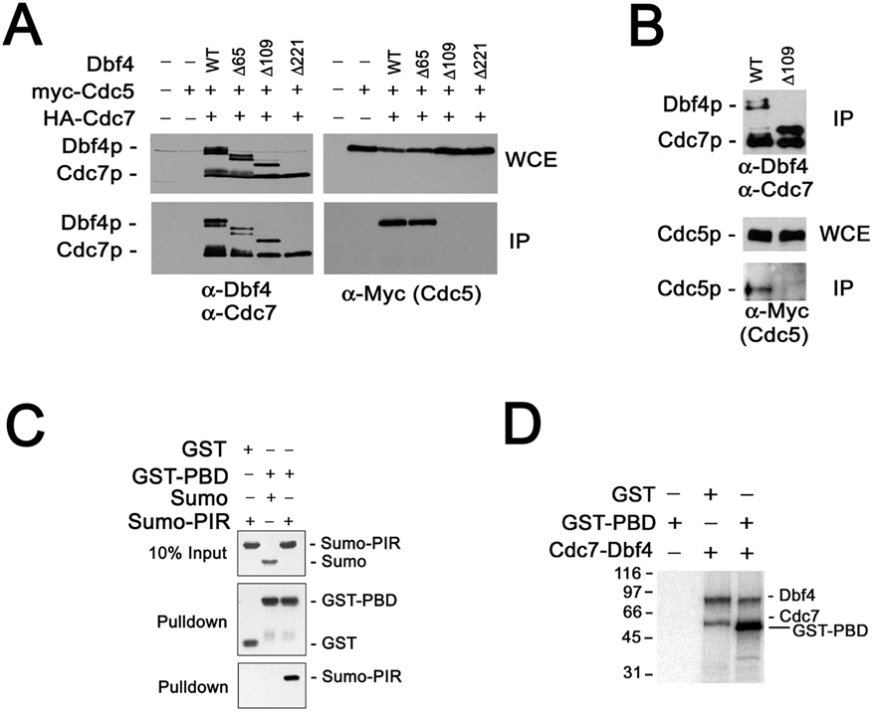
Dbf4p residues 67–109 interact directly with the Polo PBD. (A) HA-Cdc7p-Dbf4p kinase or HA-Cdc7p plus Dbf4p truncation mutants were expressed in Sf9 cells together with Polo and immunoprecipitated using 12CA5 antibody. Blots were probed with anti-Myc (Cdc5p), anti-Cdc7p and anti-Dbf4p polyclonal antibodies. (B) Endogenous HA3-Cdc7p-Dbf4p complexes were immunoprecipitated using 12CA5 antibody from *HA3-CDC7 DBF4 CDC5-Myc15* (M2741) and *HA3-CDC7 dbf4-NΔ109 CDC5-Myc15* (M2743) yeast strains following nocodazole arrest and probed for Cdc7, Dbf4, and Cdc5-Myc. (C) Purified Sumo and Sumo-Dbf4_67–109_ proteins were co-incubated with purified GST-PBD or GST alone; proteins were pulled down using glutathione-Sepharose beads and blotted with antibodies against GST or Sumo. (D) *In vitro* phosphorylation of GST-PBD using purified Cdc7p-Dbf4p kinase.

Dbf4p binds and activates the Cdc7p kinase subunit in yeast and has no known role apart from its interaction with Cdc7p [14],[60]. To determine whether the interaction between Dbf4p and Polo occurred in the context of the full-length Cdc7p-Dbf4p kinase, Sf9 cells were co-infected with baculoviruses expressing Polo, wild type HA-Cdc7p-Dbf4p or wild type HA-Cdc7p with various Dbf4p deletion derivatives. HA-Cdc7p-Dbf4p kinase was immunoprecipitated using an antibody against the HA tag and examined for the presence of Polo. All the Dbf4p deletion derivatives we examined interact with Cdc7p and activate normal Cdc7p kinase activity ([26] and data not shown). Whereas Polo interacted with full-length Cdc7p-Dbf4p and Cdc7p-Dbf4-NΔ65p, Polo did not interact with Cdc7p-Dbf4p complexes that lacked the Dbf4p N-terminal 109 residues required for the Polo two-hybrid interaction (Figure 2A). These data indicate that full length Cdc7p-Dbf4p kinase interacts with Polo but that Dbf4p residues 65–109 are required for this interaction. Importantly, HA-Cdc7p-Dbf4p interacts with Cdc5p in yeast when the proteins are expressed at endogenous levels and this interaction also depends on the Dbf4p N-terminus (Figure 2B).

We next tested whether Polo bound directly to Dbf4p using purified proteins. GST-PBD and Sumo-Dbf4p_67–109_ fusion proteins purified from *E. coli* were mixed, pulled down using glutathione-Sepharose and analyzed by immunoblotting. Although Sumo alone did not interact with GST-PBD, Sumo-Dbf4p_67–109_ interacted with GST-PBD but not with GST alone (Figure 2C). These data indicate that Dbf4p residues 67–109 (that we refer to as the Dbf4p PIR) are sufficient for a direct interaction with the Polo PBD.

### Dbf4p Inhibits Polo Activity

Cdc7p-Dbf4p is required to initiate DNA replication, but is present throughout S-phase and during the metaphase to anaphase transition. Dbf4p is subject to APC-Cdc20p dependent degradation [16] but some protein is still present in late mitotic mutants that have activated the Cdc20p but not the Cdh1p form of the APC [17]. We examined Dbf4 protein abundance relative to Pds1 protein in cells moving synchronously through the cell cycle, since Pds1p is degraded at the onset of anaphase by APC-Cdc20 [61]. We found that although the abundance of both proteins declines at the same time, Pds1p is absent during mitosis while some fraction of Dbf4p persists (Figure S5). In contrast, Dbf4p has very low abundance or is absent in cells arrested in G1-phase by mating-pheromone when the APC-Cdh1p is active [12],[13],[15],[17] and Dbf4p is stabilized by inactivation of the APC in G1 or by removal of its N-terminal D-box [13], [15]–[17]. Together these data suggest that Dbf4p degradation can occur via both APC-Cdc20p and APC-Cdh1p mediated ubiquitylation.

**Figure 3:**
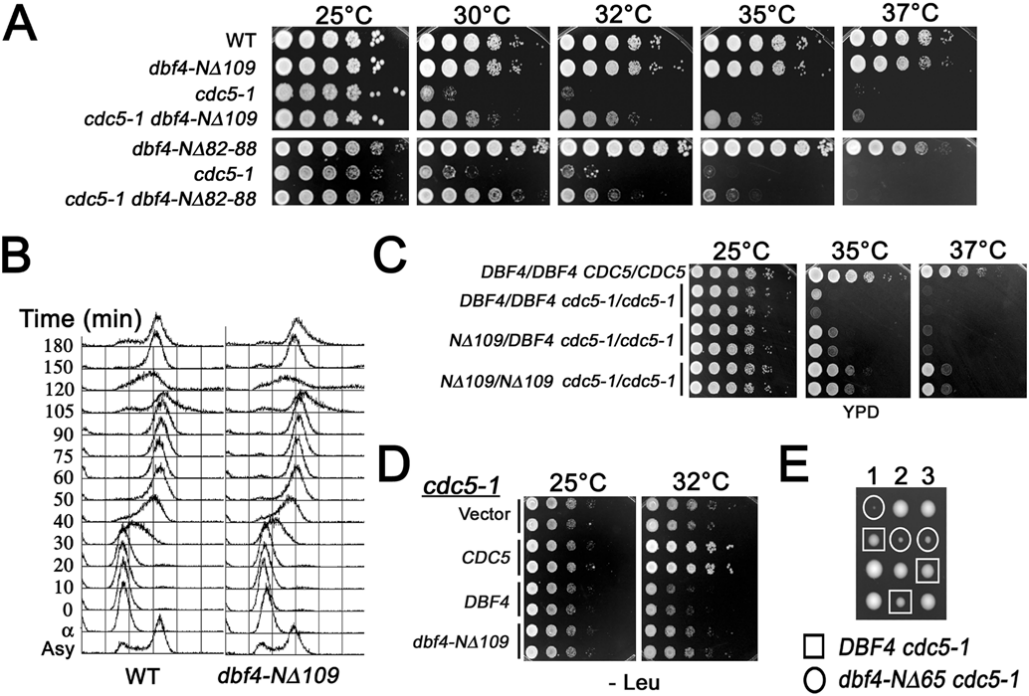
The Dbf4p N-terminus inhibits Polo activity. (A) *dbf4-NΔ109* rescues the *cdc5-1* temperature sensitivity. Indicated strains were spotted at ten-fold serial dilution on YPD and grown at increasing temperatures. (B) Cell cycle progression of wild type and *dbf4-NΔ109* at 30°C by flow cytometry of alpha-factor arrested (t = 0) and released cells (t = 10 to 180 mins). (C) The indicated diploid strains were spotted at increasing temperatures (D) *DBF4 cdc5-1* was transformed with the indicated *ARS CEN* plasmids and spotted at 25°C and 32°C (E) Representative tetrads from a *dbf4-NΔ65* (M2007) cross to *cdc5-1* (M1614).

Since budding yeast Polo is not required for DNA replication but promotes multiple mitotic activities, we reasoned that Cdc7p-Dbf4p might influence Polo activity during mitosis. The *dbf4-NΔ109* mutant progresses normally through the cell cycle and does not exhibit any obvious growth defects or temperature sensitivity (Figure 3A, B) [26]. We therefore tested for genetic interactions between *dbf4-NΔ109* and the *cdc5-1* temperature sensitive (ts) mutant. At the restrictive temperature *cdc5-1* cells arrest in late telophase with divided nuclei, elongated spindles and high Cdk1p-Clb2p levels indicating a failure to exit mitosis [34],[35],[62]. The *cdc5-1* mutant is ts at 30°C on rich media, but we found that *dbf4-NΔ109* suppressed the *cdc5-1* temperature sensitivity up to 35°C indicating a strong suppression of its growth defect (Figure 3A). The *dbf4-Δ82–88* mutant defective for interaction with Polo also suppressed the *cdc5-1* ts (Figure 3A). Since the *cdc5-1* ts suppression was reduced in heterozygous *dbf4-NΔ109*/*DBF4* diploids compared to *dbf4-NΔ109*/*dbf4-NΔ109* diploids, i.e. *dbf4-NΔ109* was haploinsufficient (Figure 3C), our data strongly suggests that Dbf4p is a Polo inhibitor and that loss of the Dbf4-Polo interaction leads to increased *cdc5-1* activity.

**Figure 4:**
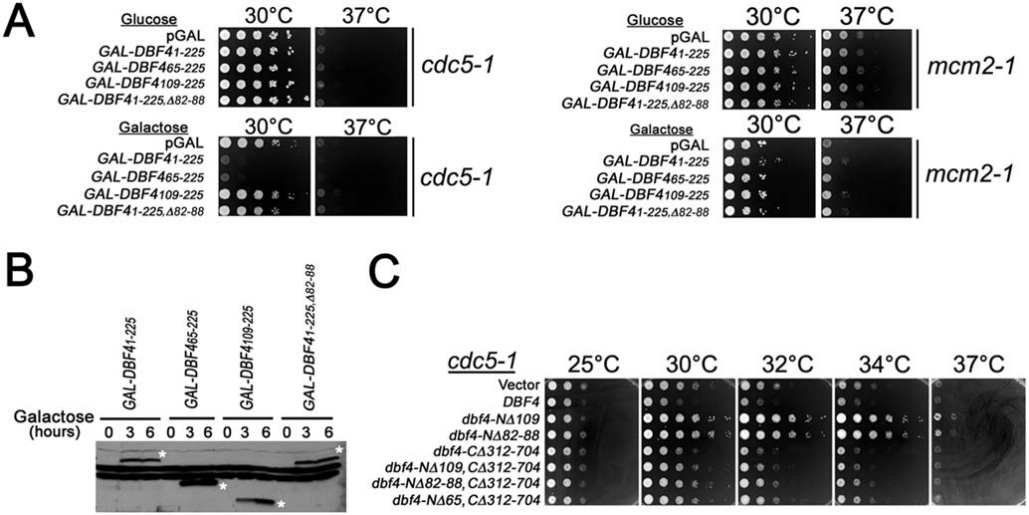
Dbf4p is a Polo inhibitor. (A) *cdc5-1* and *mcm2-1* strains expressing the indicated Dbf4 N-terminal regions from the *GAL1* promoter were plated onto glucose (repressing conditions) or galactose to induce Dbf4p expression. (B) Dbf4p Western blot of total cell extracts induced for *GAL1*-*DBF4* expression at 0, 3, and 6 hours. Asterisks indicate N-terminal Dbf4 peptides. (C) High copy vectors expressing wild type *DBF4* and mutant genes were transformed into *cdc5-1* (M1614) and spotted at 10-fold serial dilutions at the indicated temperatures.

We confirmed that Dbf4p inhibited Polo activity using several independent genetic tests. An extra plasmid copy of wild type *DBF4* but not *dbf4-NΔ109* inhibited the growth of *cdc5-1* cells (Figure 3D). A *dbf4-NΔ65* mutant that disrupts the D-box (residues 62–70) resulting in elevated protein levels was synthetically sick or lethal in combination with *cdc5-1* (Figure 3E). Since the *dbf4-NΔ65* mutant exhibits a wild type growth rate and normal S-phase entry and progression (data not shown; [26]) but binds to Polo, this suggests that elevated Dbf4p levels are deleterious to *cdc5-1* activity. Finally, elevated expression of the Dbf4p N-terminus from the *GAL1* promoter completely inhibited the growth of *cdc5-1* cells but had no effect on the growth of wild type (not shown) or a *mcm2-1* ts mutant. Mcm2 is a component of the MCM helicase, which is thought to be the physiological target of Cdc7p-Dbf4p during the initiation of DNA replication [11]. The inhibition of *cdc5-1* growth depended on the Dbf4p-Polo interaction, since deletion of the PIR (residues 66–109) or Dbf4p residues 82–88 abrogated the growth inhibition (Figure 4A, B). Since the Dbf4p N-terminus interacts with the PBD, this data suggest that overexpression of Dbf4 N-terminal peptides interferes with essential Polo-substrate interactions by competitive inhibition. Together, these data indicate that the Dbf4p N-terminus inhibits Polo activity and that this inhibition requires residues 66–109, which are also required for the Dbf4p-Polo physical interaction.

We wanted to determine whether the Cdc7p kinase subunit is required to inhibit Polo in the FEAR or MEN pathways. This is not straightforward since Cdc7p is an essential protein kinase. Importantly, inhibiting Cdc7p activity would not only inhibit replication origin firing but would also likely induce the replication checkpoint that inhibits the metaphase to anaphase transition and MEN activation [4]. Inhibiting Cdc7p activity would thus interfere with the mitotic pathways we would like to measure. Therefore, we addressed this question indirectly by taking advantage of our observation that high copy *dbf4-NΔ109* suppressed the *cdc5-1* ts phenotype (Figure 4C). Since Dbf4p residues required for interaction with Cdc7p map between residues 312–704 (C.G. and M.W. unpublished data), Dbf4-NΔ109 protein (expressed in high copy) will compete with full length Dbf4p (in single copy) for Cdc7p binding. Therefore, our finding suggested that high copy expression of Dbf4-NΔ109p reduced the cellular concentration of wild type Cdc7p-Dbf4p, the likely Cdc5p inhibitor, which suppressed the *cdc5-1* ts allele (Fig 4C). High copy expression of Dbf4-Δ82–88p that does not interact with Polo also suppressed the *cdc5-1* ts (Fig 4C). Importantly, deleting Dbf4p C-terminal residues required for interaction with Cdc7p (CΔ312–704) eliminated the ts suppression by high copy *dbf4-NΔ109* and *dbf4-Δ82–88*. These data are consistent with full-length Cdc7p-Dbf4p kinase acting as the physiological Polo inhibitor.

### Cdc7p-Dbf4p Phosphorylates the PBD

Wild type Cdc7p-Dbf4p might inhibit Polo abundance or kinase activity during the cell cycle and thus explain our genetic data. However, we saw little difference in Polo protein levels, cell cycle expression or Polo kinase activity comparing wild type yeast with the *dbf4-NΔ109* mutant (Figure S2). This suggests that Dbf4p inhibits Polo independently of altering its expression or kinase activity. This is consistent with our genetic data since loss the Dbf4p PIR suppresses the *cdc5-1* allele yet the Cdc5-1 protein retains considerable protein abundance and kinase activity at the non-permissive temperature [63]. The mitotic exit defect associated with *cdc5-1* is due to a single P511L amino acid substitution preceding polo-box 1 of the PBD [64], strongly suggesting that the *cdc5-1* growth defect is caused by a defect in substrate recognition. Since genetically *DBF4* is a negative *CDC5* regulator we hypothesized that Cdc7p-Dbf4p phosphorylates Polo to prevent its access to key substrates in the MEN. Consistent with this possibility, we found that purified Cdc7p-Dbf4p phosphorylated recombinant GST-PBD but not GST alone (Figure 2D).

### Dbf4p Inhibits Cdc14p Nucleolar Release

**Figure 5:**
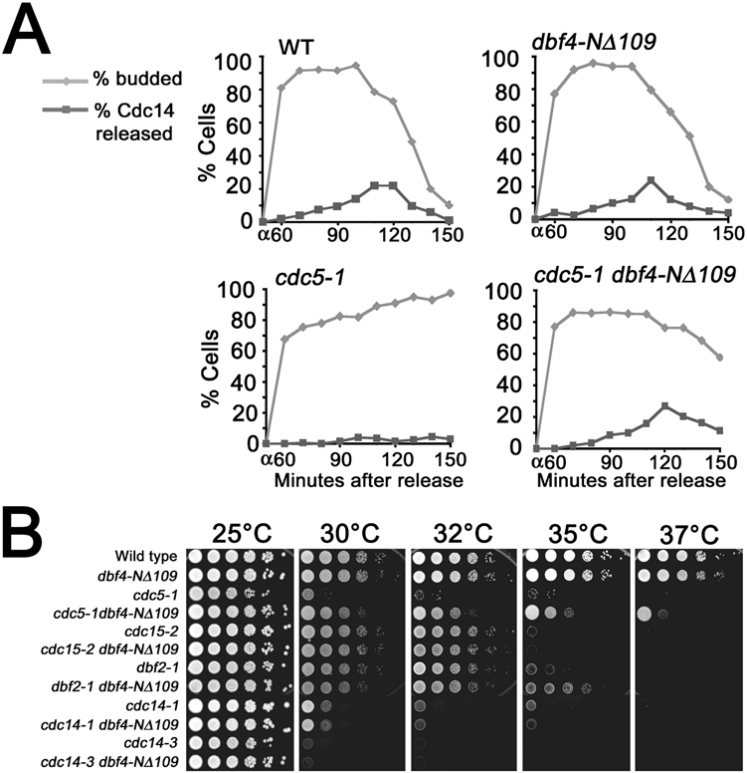
*DBF4* inhibits Cdc14p nucleolar release and the MEN pathway. (A) Deletion of the Dbf4p PIR rescues Cdc14p nucleolar release at high temperature. M1992, M2005, M2139 and M2287 were arrested with alpha-factor at 25°C and released into YPD at 34°C followed by alpha-factor re-addition at 60 minutes. Entry and exit from the cell cycle was analyzed as a percentage of budded cells. Cdc14-EGFP release was analyzed in at least 100 cells at each time point and is reported as a percentage of cells with diffuse Cdc14p-EGFP nuclear staining (absence of nucleolar Cdc14p). (B) *dbf4-NΔ109* rescues the growth defect of the *cdc5-1* and *dbf2-1* strains at 35°C. Single and double mutant strains were spotted at 10-fold dilutions on YPD and incubated at increasing temperatures for 2 to 3 days.

The Cdc14p phosphatase is sequestered within the nucleolus during the cell cycle prior to FEAR and MEN pathway activation [50],[65]. Activation of the FEAR pathway allows limited Cdc14p nucleolar release, which promotes rDNA and telomere segregation during early anaphase [42], [44]–[46]. Cdc14p is then fully released by MEN activation and antagonizes Cdk activity to trigger exit from mitosis [66]. Since the *cdc5-1* mutant fails to release Cdc14p at the restrictive temperature [66], suppression of the *cdc5-1* ts by deletion of the Dbf4p PIR (Figure 3A) suggested that Cdc14p release is likely restored in these cells at higher temperatures. We quantitated the nucleolar release of Cdc14-EGFP in wild type, *dbf4-NΔ109*, *cdc5-1* and *cdc5-1 dbf4-NΔ109* cells at a restrictive temperature for *cdc5-1*. Cells were arrested in G1-phase at the permissive temperature and then released into the cell cycle at 34°C. The *dbf4-NΔ109* cells progressed through the cell cycle and released Cdc14p similarly to wild type cells (Figure 5A). Consistent with previous reports, the *cdc5-1* mutant failed to release Cdc14p from the nucleolus but a significant amount of Cdc14p was released from the nucleolus in the *cdc5-1 dbf4-NΔ109* mutant (Figure 5A). We noticed a delay in mitotic progression at 34°C in the *cdc5-1 dbf4-NΔ109* cells evidenced by a somewhat longer duration of Cdc14p release compared to the wild type and delayed cytokinesis (indicated by the delayed appearance of unbudded (G1) cells). This data indicates that Dbf4p inhibits Polo activity to prevent Cdc14p release, which might be significant during a slowed S-phase or during periods of replication stress.

### Dbf4p Also Inhibits the MEN Kinase Dbf2p

Inactivation of Bfa1p-Bub2p is required to activate the MEN [37]. This signaling cascade is partially activated as a result of Bfa1p phosphorylation by Polo, which leads to activation of the Cdc15p and Dbf2p-Mob1p kinases (reviewed by [47]). Dbf2p kinase activation requires a Bub2p-Bfa1p independent function of Polo as well [49], indicating that Polo either directly promotes Dbf2p kinase activity or promotes a MEN-independent pathway that activates Dbf2p. We tested whether Dbf4p functions as a negative regulator of MEN activation by examining whether deletion of the Dbf4p PIR could suppress growth defects associated with additional ts mutants in the MEN (Figure 5B). We examined the growth of double mutants of *cdc5-1*, *cdc15-2*, *dbf2-1*, *cdc14-1*, or *cdc14-3* with *dbf4-Δ109*. As with *cdc5-1*, loss of the *DBF4* PIR rescued the growth of *dbf2-1* cells at the non-permissive temperature. In contrast, the *dbf4-Δ109* mutant failed to suppress the ts phenotype of *cdc15-2*, *cdc15-4* (not shown), *cdc14-1*, or *cdc14-3* mutants (Figure 5B). This suggests that Dbf4p may specifically inhibit Polo activation of Dbf2p and not Polo inactivation of Bub2p-Bfa1p GAP activity. Taken together, our observations suggest that Dbf4p antagonizes Polo activation of some Bfa1p-Bub2p independent step in MEN activation. This interpretation is further supported by the following experiments.

### Dbf4p Regulates a Bfa1p-Bub2p Independent Polo Activity during Mitotic Exit

**Figure 6:**
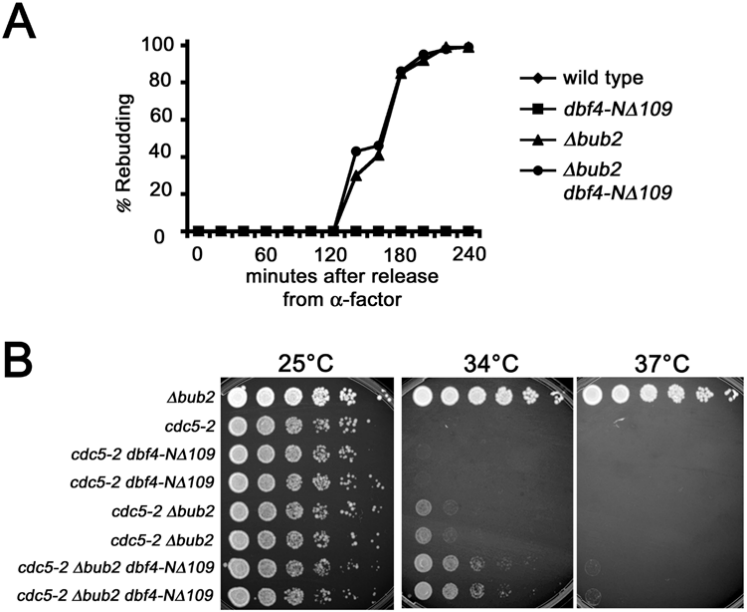
*DBF4* regulates mitotic exit independently of *BFA1*-*BUB2*. (A) Deletion of the *DBF4* polo-box interacting region does not cause rebudding. W303-1A, M1652, M1656 and M1860 were synchronized with alpha-factor and released into YPD containing 15 ug/ml of nocodazole at 30°C. Samples were quantitated for the percentage of rebudded cells (large budded cells with a new bud). (B) Deletion of *BUB2* and the *DBF4* PIR cooperate to suppress the growth defect of *cdc5-2*. Strains of the indicated genotypes were spotted at 5-fold serial dilutions on YPD and grown at increasing temperature. Two isolates are shown for the *cdc5-2* recombinants.

Deletion of *BUB2* (or *BFA1*) is sufficient to cause premature mitotic exit when cells are arrested in metaphase with the spindle poison nocodazole [67],[68]. This causes large budded cells to exit mitosis and rebud in the absence of chromosome segregation or cytokinesis. Since Dbf4p is a negative regulator of Polo activity, we examined whether deletion of the Dbf4p PIR was sufficient to induce rebudding in the presence of spindle poisons. Cells were arrested in G1, released into media containing nocodazole and quantitated for rebudding (Figure 6A). In contrast to *bub2Δ*, *dbf4-NΔ109* did not allow rebudding in a wild type background nor did this mutation advance rebudding in a *bub2Δ* background. This indicated that loss of the Dbf4p Polo interaction is not sufficient to cause mitotic exit during metaphase and suggested that Dbf4p Polo inhibition may act independently of Bub2p-Bfa1p.

Null alleles of *CDC5* fail to activate the MEN and arrest in telophase with unphosphorylated Bfa1 [37]. Although the *cdc5-1* mutant is also defective in MEN activation it is proficient for Bfa1p phosphorylation and retains substantial Polo kinase activity at the non-permissive temperature (NPT) [37],[63]. This suggests that the *cdc5-1* mutant is defective in activating a Bfa1p-independent function of the MEN, perhaps in FEAR pathway activation or activating a downstream MEN target. In contrast to *cdc5-1*, *cdc5-2* cells neither phosphorylate Bfa1p nor activate the MEN at the NPT [37]. The *cdc5-2* temperature sensitivity is partially rescued by deletion of either *BFA1* or *BUB2* [37] but not by *dbf4-NΔ109* (Figure 6B). However, in a *bub2Δ* the *cdc5-2* temperature sensitivity was further suppressed by deletion of the Dbf4p PIR. So, eliminating the requirement for Bfa1p-Bub2p inactivation in *cdc5-2* cells (i.e. *cdc5-2 bub2Δ*), allowed *dbf4-NΔ109* to further suppress the *cdc5-2* ts and promote mitotic exit at 34°C (Figure 6B). These data are consistent with the interpretation that Dbf4p primarily inhibits Polo activation of a Bub2p-Bfa1p *independent* step in MEN activation, e.g. Dbf2p activity, Cdc14p release in the FEAR pathway, or some unknown activity.

### *DBF4* Prevents Mitotic Exit When the Nucleus Is Mispositioned

In the budding yeast, *KAR9* and *DYN1* encode cytoplasmic microtubule-associated and motor proteins, respectively, that operate in two redundant pathways essential for the correct positioning of the nucleus during anaphase (reviewed by [7]). Deletion of either gene results in a small percentage of cells with nuclear orientation defects but deletion of both genes is lethal. In response to nuclear misorientation, *S. cerevisiae* inhibits premature activation of the MEN via the spindle position checkpoint (SPOC). When the SPOC is activated the Kin4p kinase localizes to spindle pole bodies (SPB) to counteract Polo Bfa1p phosphorylation [8],[9],[55]. Kin4p thus counteracts Polo inactivation of the Bfap1-Bub2p GAP and this inhibits MEN activation. Failure to adequately respond to nuclear mispositioning allows inappropriate nuclear division within the mother cell, leading to anueploidy and loss of viability. Given that Polo Bfa1p phosphorylation is prevented when the SPOC is activated, we hypothesized Dbf4p may also inhibit Polo to prevent mitotic exit in response to nuclear misorientation.

**Figure 7:**
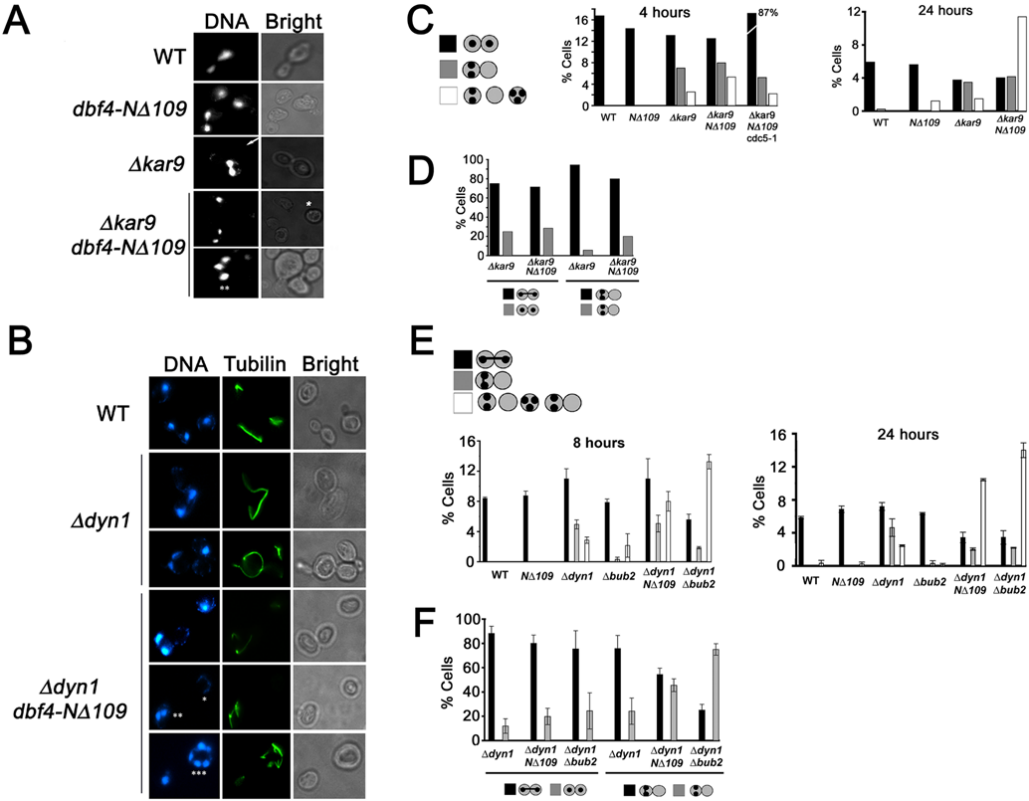
Deletion of the *DBF4* PIR allows exit from mitosis in cells with misoriented nuclei. (A, B) Representative cells showing nuclear positioning and mitotic arrest defects. Nuclear position was analyzed after DAPI staining. Cells with anaphase spindles located exclusively in the mother cell body are noted (white arrow). Anucleate (*), binucleate (**) and multinucleate (***) cells were frequently observed in *kar9Δ dbf4-NΔ109* (A) and *dyn1Δ dbf4-NΔ109* (B) cells. (C, E) Quantitation of nuclear segregation defects. >300 cells were counted for each strain and nuclear position was represented as a percentage of the total cells in culture. Black bars represent cells with nuclei segregated between mother and daughter cells; gray bars, divided nuclei in the mother cell; white bars, multinucleate and anucleate cells. (D, F) Spindle morphology of strains with misoriented nuclei and spindles. >50 cells were counted for each genotype. Black bars represent cells with mitotic spindles. Gray bars represent cells that have exited mitosis.

To test whether *DBF4* inhibited mitotic exit when nuclei were mispositioned, we examined wild type, *dbf4-NΔ109*, *kar9Δ* and *kar9Δ dbf4-NΔ109* strains for evidence of mitotic exit in the presence of mispositioned nuclei. Asynchronous cultures were grown at 25°C and shifted to 30°C for 4 or 24 hours prior to analysis to increase the penetrance of the nuclear mispositioning phenotype. Although wild type and *dbf4* mutant cells did not misorient their nuclei (Figure 7C), both *kar9Δ* and *kar9Δ dbf4-NΔ109* strains had approximately equal number of cells with nuclear positioning defects. Importantly, deletion of the Dbf4p PIR resulted in a 2 to 3-fold increase (to 6%) in binucleate, anucleate and multinucleate cells at 4 hours (Figure 7A, C, D), which was *CDC5*-dependent, suggesting that premature mitotic exit occurred. At 24 hours the number of aberrant chromosome segregation events due to loss of the Dbf4p PIR increased six-fold (12%) relative to *kar9Δ* alone (2%). Since exit from mitosis causes spindle disassembly, we quantitated the spindle morphology in cells containing correctly segregated nuclei (between the mother and daughter cells) and in those cells where anaphase had initiated solely within the mother cell. Although *kar9Δ* and *kar9Δ dbf4–NΔ109* cells had similar frequencies of intact or disassembled spindles when nuclear division proceeded normally, *kar9Δ dbf4-NΔ109* cells showed a three-fold increase in spindle disassembly within the mother cell (leading to a bi-nucleate mother cell) compared to *kar9Δ* single mutants at 4 hours (Figure 7D). These data indicate that mitotic exit occurs in these cells in the absence of the Dbf4p PIR.

Similarly, we tested whether deletion of the Dbf4p PIR allowed premature mitotic exit in cells disrupted in the dynein pathway (Figure 7B, E, F). The spindle-positioning defect associated with deletion of *DYN1* is especially prominent at low temperatures. Although after 8 hours at 14°C only ∼3% of *dyn1Δ* cells had exited mitosis inappropriately, deletion of the Dbf4p PIR resulted in a 3- to 4-fold increase in mitotic exit as evidence by the appearance of multinucleate and anucleate cells (Figure 7E) and this frequency was increased at 24 hours. For comparison, a *dyn1Δ bub2Δ* strain had a similar but higher frequency of segregation defects (Figure 7E, F). Therefore, the bypass of the SPOC following loss of the Dbf4p-Polo interaction is comparable to deletion of the MEN inhibitor *BUB2*. As with the *kar9Δ* mutant, we observed that a higher percentage of *dyn1Δ dbf4-NΔ109* cells compared to *dyn1Δ* single mutants that divided nuclei within the mother cell had disassembled their spindles (Figure 7F). These observations indicate that deletion of the Dbf4p N-terminus (including the PIR) overrides the mitotic arrest normally activated by the SPOC.

## Discussion

We found that Dbf4p inhibited Polo kinase during mitosis very likely through a direct interaction with the polo-box domain. This interaction inhibited MEN pathway activation, nucleolar release of the Cdc14p phosphatase and was likely critical to maintain genome integrity during activation of the spindle position checkpoint. These results therefore have important implications for understanding Dbf4p function and the regulation of mitotic progression in eukaryotic cells.

### Defining the Interaction between Dbf4p and Polo

The N-terminal third of Dbf4p encodes multiple functions: a destruction box (residues 62–70), two putative nuclear localization signals (residues 55–61, 251–257) and a BRCT-like domain (residues ∼117–220). Nonetheless the 265 N-terminal amino acids of Dbf4p are not essential as long as a nuclear localization signal is present [26]. Deletion of the Dbf4p N-terminus through the PIR has no observable effect on growth, viability, or cell cycle progression either under normal growth conditions or in the presence of replication or spindle poisons ([26] and data not shown). Here, we discovered an interaction between Dbf4p and the Polo PBD that mapped to a short sequence of ∼40 amino acids preceding the BRCT-like domain. Although a two-hybrid interaction between Polo and Dbf4p was reported before [39], the significance of this interaction was not determined.

The Polo PBD functions as a module for binding phosphorylated proteins and thereby targets Polo to its cellular substrates [57],[59]. The question naturally arises as to whether phosphorylation of the Dbf4p PIR is required for PBD binding. Currently, our observations suggest that phosphorylation is not required. A polo-box binding consensus sequence (S(pS/pT)P/X) is not present within this region of Dbf4p [59] and mutation of all putative serine and threonine residues within the Dbf4p PIR did not significantly diminish the interaction in the two-hybrid assay. These data suggest that phosphorylation of Dbf4p is not crucial for Polo binding. Our observation that the Dbf4p PIR purified from *E. coli* directly interacted with the Polo PBD also supports this notion. Thus phosphorylation of Dbf4p was not critical for binding to Polo *in vitro*, but we cannot exclude the possibility that phosphorylation contributes to Dbf4p-Polo binding *in vivo* when the two proteins are present at physiological concentrations.

### When Does Dbf4p Inhibit Polo and Mitotic Exit?

The finding that deletion of the Dbf4p PIR significantly suppressed the ts phenotype of *cdc5-1* suggests that Cdc7p-Dbf4p inhibits Polo during the normal cell cycle and perhaps during periods of replication stress, when Cdc7p-Dbf4p is stabilized [17]. Our data clearly demonstrate a role for Dbf4p in inhibiting mitotic exit, since loss of the Dbf4p PIR suppressed both *cdc5* and *dbf2* ts mutants and allowed sustained Cdc14p phosphatase release and cytokinesis in the *cdc5-1* mutant at the NPT. However, during an unperturbed cell cycle the absence of this regulation had little impact. This is likely attributable to the fact that the cell cycle regulation of Polo activity is complex and modulated by multiple cell cycle checkpoints. Since *dbf4-NΔ109 bub2Δ* double mutants were more sensitive to growth on spindle poisons than either mutant alone (Figure S3), the Dbf4p-Polo and Bfa1p-Bub2p pathways may work together to suppress premature activation of the mitotic exit network.

It was shown very recently that increased expression of a non-destructible form of Dbf4p (Dbf4-NΔ65p) could delay rDNA segregation when Clb5p was also stabilized [16]. This raises the possibility that Dbf4p inhibits Cdc14p release via the FEAR pathway under some circumstances and is consistent with our data showing that Dbf4p is a Polo inhibitor. However, we found no evidence that the *dbf4-NΔ109* allele promoted premature rDNA segregation (a FEAR pathway event) in the *cdc5-1* mutant (Figure S4). Similarly, we found no evidence that *dbf4-NΔ109* caused premature Cdc14p release in a strain deleted for *FOB1*, which is thought help sequester Cdc14p in the nucleolus (Figure S4). These data suggest that Dbf4p is not specifically inhibiting the FEAR pathway.

The budding yeast SPOC prevents premature exit from mitosis in part by inducing Kin4p phosphorylation of Bfa1p, which antagonizes the Polo-dependent inhibition of the Bfa1p-Bub2p GAP [8],[9],[55]. Premature exit from mitosis in *dbf4-NΔ109 dyn1* and *dbf4-NΔ109 kar9* double mutants suggests that Dbf4p regulation of Polo is critical for robust cell cycle control in response to nuclear mispositioning. What remains unclear however, is whether this Dbf4p activity is regulated following activation of the SPOC. For instance, Cdc7p-Dbf4p may inhibit Polo to buffer against premature release of Cdc14p during late S-phase or early M-phase whether or not the SPOC is activated. Therefore, in the absence of the Dbf4p-Polo regulation Polo may prematurely activate the MEN before a nuclear orientation defect is sensed by the SPOC. Following APC-Cdc20p and APC-Cdh1p activation during anaphase onset and exit, we suggest that degradation of Dbf4p provides a positive feedback loop for full Polo activation of the MEN and ultimately, cytokinesis.

### How Does Dbf4p Inhibit Polo and Mitotic Exit?

Dbf4p did not influence Polo protein levels or overall kinase activity. Dbf4p nonetheless inhibited Polo activity since *dbf4* mutants unable to interact with Polo significantly suppressed the *cdc5-1* temperature sensitivity. Since the *cdc5-1* allele retains significant Polo protein expression, Bfa1p phosphorylation and overall Polo kinase activity at the non-permissive temperature [37],[63], the primary *cdc5-1* MEN defect is in a Bfa1p-independent requirement for MEN pathway activation, perhaps Dbf2p activation or some other MEN-dependent step. Our observation that the *dbf2-1* temperature sensitivity was also suppressed by loss of the Dbf4p PIR suggests that Dbf4p may specifically inhibit Polo activation of Dbf2p kinase independently of Bfa1p-Bub2p phosphorylation. Dbf2p was recently shown to promote cytoplasmic Cdc14p localization following MEN activation [69]. In addition, deletion of both *BUB2* and the *DBF4* PIR suppressed the *cdc5-2* ts better than either single mutant alone. In other words, since *dbf4-NΔ109* further suppressed the ts phenotype of a *cdc5-2 bub2Δ* strain, this supports the contention that Dbf4p regulates MEN activity independently of Bfa1p-Bub2p. Deletion of the Dbf4p PIR also did not allow rebudding in the presence of spindle poisons as seen in *bub2Δ* mutants, again suggesting that Dbf4p plays a minor or redundant role to inhibit Polo phosphorylation of Bfa1p-Bub2p.

Cdc7p-Dbf4p kinase phosphorylated the Polo PBD *in vitro* suggesting that Cdc7p-Dbf4p may antagonize Polo substrate binding. This possibility is consistent with the requirement for the PBD for targeting Polo to sites of MEN activity. Polo, Cdc15p, Dbf2p and Bfa1p-Bub2p are localized to SPB prior to activation of the mitotic exit network [47]. Thus, we favor a model whereby Cdc7p-Dbf4p kinase inhibits precocious Polo binding to critical MEN substrate(s) by phosphorylating the Polo PBD. It will be interesting to investigate whether the Cdc7p-Dbf4p inhibition of Polo is regulated by cell cycle checkpoints and to determine the precise activity of Polo that is affected.

## Materials and Methods

### Construction of Yeast Strains, Plasmids, and Baculoviruses

Strains and plasmids used in this study are listed in Tables S1 and S2, and supplemental methods in the Text S1 file. PJ69-4a cells (*MAT* **a** *trp1-901 leu2-3*,*-112 ura3-52 his3-200 gal4Δ gal80Δ LYS2::GAL1-HIS3 GAL2-ADE2 met::GAL7-lacZ*) were used for two-hybrid experiments. All other strains were derivatives of W303 (*MAT* **a** *ade2-1 trp1-1 can1-100 leu2-3*, *-112 his3-11*, *-15 ura3-1*). Construction of Dbf4p N-terminal truncation mutants was previously described [26]. Cdc14-EGFP was constructed as described [70]. *bub2Δ* strains were created by replacement of the *BUB2* ORF via homologous recombination with *Sac*I-*Cla*I *bub2Δ::URA3* fragment from pTR24 (A. Hoyt). The *BAR1* ORF was deleted by homologous recombination with linearized pZV77 containing *bar1::LEU2* (B. Futcher). Construction of *KAR9* and *DYN1* deletions were previously described [8].

For yeast two-hybrid analyses, a Gal4 DNA binding domain (GBD) fusion to Dbf4p_67–227_ was constructed by PCR amplification of Dbf4p residues 67–227 (*Nco*I-*Pst*I) and cloned into pGBKT7 (Clonetech). Deletion of 71 bp within the *ADH1* promoter sequence of pGBKT7 (-647 to -717 from the ATG) removed a Rap1p binding site and reduced the strength of GBD-Dbf4p_67–227_ expression (which was otherwise lethal) to give pCG60. Point mutations and deletions were generated by site-directed mutagenesis using the QuikChange system (Stratagene). For all mutations, the entire coding sequence was verified by DNA sequencing. Construction of baculovirus plasmids encoding WT, NΔ65Dbf4p, NΔ109Dbf4p, NΔ221Dbf4p and HA-Cdc7p was previously described [26]. The baculovirus transfer plasmid containing 3Myc-NΔ65Polo was constructed in pAcSG2 (BD Biosciences). High-titer baculoviruses were generated by transfection of Sf9 cells using the BaculoGold kit (BD Biosciences) followed by plaque purification and virus amplification.

For *in vitro* interaction assays, DNA encoding Polo amino acids 357–705 were PCR amplified with *Bam*HI-*Xma*I linkers and cloned into pGEX-KG for expression of GST-Polo_PBD_ in *E. coli*. The region encoding Dbf4p amino acids 66–109 was PCR amplified from pMW489 with *Bsa*I-*Bam*HI linkers and cloned into pSUMO (LifeSensors Inc.) for expression of Sumo-Dbf4p_67–109_.

### Growth Media and Cell Cycle Experiments

Cells were cultured in YPD (1% yeast extract, 2% bacto peptone, 2% glucose). Synchronous G1 cultures were obtained after addition of 5 µg/ml (0.1 µg/ml in *bar1Δ* cells) alpha-factor to cells for 3 hours. DNA content was analyzed by flow cytometry as previously described [17]. Drugs were added directly to plates immediately before pouring.

### Two-Hybrid Experiments

PJ69-4a cells containing pCG60 were transformed with a *S. cerevisiae* two-hybrid library. Interacting clones were recovered on medium lacking tryptophan, leucine, and histidine but containing 2 mM 3-aminotriazole at 30°C. Positive interactors were streak-purified and also tested for *ADE2* reporter activity. Prey plasmids that activated both *HIS3* and *ADE2* expression were confirmed by retransformation in PJ69-4a and then sequenced. To quantify two-hybrid interactions, co-transformed cells were spotted at ten-fold serial dilutions on selective media and grown for 2–3 days.

### Yeast Whole-Cell Extract Preparation, Immunoprecipitation, and Blotting

Yeast protein extracts were prepared for Western blotting by trichloroacetic acid extraction [71] or for immunoprecipitation (IP) by bead-beating in NP-40 lysis buffer (20 mM Tris-HCl, 150 mM NaCl, 0.5% NP-40 and 1 mM EGTA). HA-tagged proteins were immunoprecipitated using anti-HA monoclonal antibody (12CA5) conjugated to protein A-Sepharose. Blots were probed in phosphate-buffered saline containing 0.1% Tween and 1% dried milk. 12CA5 (1∶1000) was used to detect HA-tagged proteins, 9E10 (1∶1000) to detect Myc-tagged proteins, and polyclonal sera against Cdc7p (1∶4000) and Dbf4p (1∶1000) were used to detect those proteins.

### Co-Immunoprecipitation from Sf9 Insect Cells

Sf9 cells were co-infected with HA-Cdc7p, 3Myc-NΔ65Polo and Dbf4p derivatives and then immunoprecipitated as previously described [26]. Whole cell extracts and IPs were probed with polyclonal antibodies against Cdc7p (1∶4000) and Dbf4p (1∶1000) as described above. 3Myc-NΔ65Polo was probed with 9E10 monoclonal antibody against Myc (1∶1000).

### Protein Expression, Purification, and GST-Pull Down

Cdc7p-Dbf4p kinase was purified as described [17]. GST or GST-PBD was induced in BL21 cells for 3 hours at 37°C using 0.5 mM IPTG. Cells were sonicated in PBS containing 1% Triton X-100 and GST proteins were purified from soluble extracts by binding to glutathione-agarose (Amersham), elution in (20 mM Tris-HCl, 150 mM NaCl, 1 mM EDTA and 10% glycerol) containing 5 mM glutathione followed by dialysis against the same buffer. 6His-tagged Sumo and Sumo-Dbf4p were expressed in BL21 cells and extracted in HEPES extraction buffer (50 mM HEPES-KOH, pH 7.5, 150 mM NaCl, 2 M Urea and 10% glycerol). Proteins were loaded onto a Ni++ column and washed (20 mM HEPES-KOH, pH 7.5, 200 mM NaCl and 10% glycerol) before elution using an imidazole gradient. For GST pull-downs, Sumo, Sumo-Dbf4p, GST and GST-Polo were incubated with glutathione-agarose in the presence of buffer (20 mM Tris-HCl pH 7.0, 300 mM NaCl, 0.5% NP-40 and 1 mM EGTA) for 1 hour at 4°C. The glutathione agarose beads were washed extensively and bound proteins separated on 12.5% SDS-PAGE gels. Blots were probed with polyclonal antisera raised against GST-Polo PBD (1∶1000) and yeast Smt3p (Sumo) (1∶1000).

### Kinase Assays

For *in vitro* kinase assays, purified HACdc7p-Dbf4p (100 ng) [17] was incubated with GST or GST-Polo PBD (300 ng) at 30°C in kinase buffer (50 mM Tris-HCl pH 7.5, 10 mM MgCl_2_, 1 mM DTT, 100 µM ATP and 10 µCi of [γ-^32^P] ATP) for 20 minutes. Proteins were separated on 10% SDS-PAGE and visualized by autoradiography.

### Fluorescence Microscopy

For direct fluorescence analysis of Cdc14-EGFP, cells were fixed in 3.7% formaldehyde at room temperature for 1 hour. DNA was stained using DAPI (1 µg/ml) for 10 minutes at room temperature. For the experiments in Figure 5A, the absence of a distinct Cdc14-EGFP fluorescent signal (but accompanied by diffuse nuclear and cytoplasmic fluorescence) was scored as “released.” Any cell that had a distinct Cdc14-EGFP nucleolar fluorescence was counted as sequestered. Spindle morphology was detected after spheroplasting cells and incubation in methanol/acetone prior to incubation with antibodies: rat anti-tubulin (YOL1/34 Accurate Chemicals, 1∶10) and goat anti-rat FITC (Jackson Immunoresearch, 1∶50). Cells were imaged using a 60× objective.

## Supporting Information

Figure S1 Western blot of Gal4-DNA binding domain fusions to various Dbf4 N-terminal fragments for the 2-hybrid assays shown in Figure 1.
(3.15 MB TIF)

Figure S2 Cdc7p-Dbf4p does not alter Polo kinase abundance or activity. (A) WT (K6019) and *dbf4-NΔ109* (M1874) containing *CDC5-HA3* were arrested in G1, released into the cell cycle and blotted for Cdc5-HA3 protein. Budding index is shown. (B) *DBF4 CDC5-4XGFP SPC42-eqRFP* (M2750) and *dbf4-NΔ109 CDC5-4XGFP SPC42-eqRFP* (M2748) were arrested in G1 phase with mating pheromone and released into nocodazole. These were scored for Cdc5-4XGFP localization to the spindle pole body. (C) Strains expressing Cdc5-3HA protein were arrested at 30°C with alpha-factor (α) and released into YPD containing 0.2 M hydroxyurea (HU) or 15 µg/ml nocodazole (Noc) for 2 hours. Extracts were blotted for Cdc5 protein and Cdc5p kinase activity was measured following IP (D) Quantitation of kinase activity from three independent experiments.
(0.75 MB TIF)

Figure S3 Deletion of the Dbf4p PIR causes decreased growth in the presence of spindle poisons. Indicated strains were spotted at 10-fold serial dilutions on YPD or YPD containing 10 µg/ml benomyl.
(2.47 MB TIF)

Figure S4 (A) Deletion of the Dbf4p PIR does not enhance nucleolar segregation in *cdc5-1* cells. *DBF4*, *dbf4-NΔ109*, *cdc5-1* and *cdc5-1 dbf4-NΔ109* cells transformed with a centromeric EGFP-Nop1 plasmid were arrested in G1 with mating pheromone and released into the cell cycle in YPD at 34°C. Alpha-factor was added back after budding to permit a single cell cycle. Samples were taken at the indicated time points and scored for the presence of one or two GFP signals by florescence microscopy. (B) Deletion of the Dbf4p PIR does not enhance Cdc14 release when combined with deletion of *FOB1*. *Δfob1 CDC14-EGFP* (M3149) and Δ*fob1 dbf4-NΔ109 CDC14-EGFP* (M3148) were arrested in G1 with mating pheromone and released into the cell cycle at 30°C. Alpha-factor was added back after budding to follow a single cell cycle. Samples were taken at the indicated time points and scored for release of Cdc14 from the nucleolus.
(0.22 MB TIF)

Figure S5 Some Dbf4p persists after Cdc20 activation during an unperturbed cell cycle. *PDS1-HA3 DBF4-Myc18* (M3161) was arrested in G1 with mating pheromone and released into the cell cycle at 20°C. Samples were taken at the indicated time points. Protein extracts were made for Western blotting and cells processed for DNA content analysis by flow cytometry. Western blots were probed with 9E10 α-Myc (Dbf4-Myc18) and 12CA5 α-HA (Pds1-HA) antibodies.
(3.24 MB TIF)

Table S1 Yeast strains used in this study.
(0.08 MB DOC)

Table S2 Plasmids used in this study.
(0.05 MB DOC)

Text S1 Supplementary methods: Cdc5 protein abundance, kinase activity, and SPB localization; nucleolar segregation assay; and Dbf4 cell cycle abundance.
(0.03 MB DOC)
