# Supplementary figures and images for: Cdc7p-Dbf4p Regulates Mitotic Exit by Inhibiting Polo Kinase
Source: PLoS Genet. 2009 May 29;5(5):e1000498. doi: 10.1371/journal.pgen.1000498 (PMC2682205; doi:10.1371/journal.pgen.1000498)

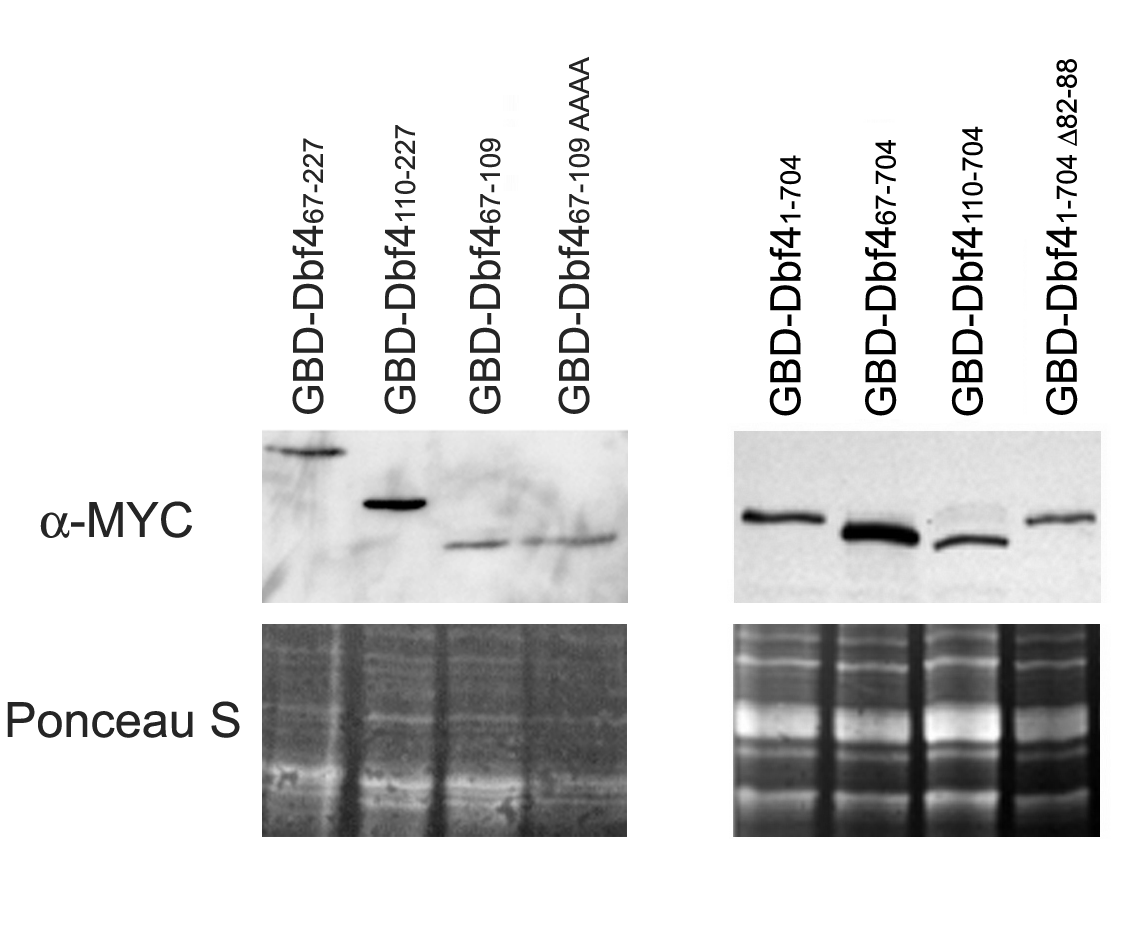

Supplement: Figure S1 — Western blot of Gal4-DNA binding domain fusions to various Dbf4 N-terminal fragments for the 2-hybrid assays shown in Figure 1. (3.15 MB TIF) [file pgen.1000498.s001.tif]

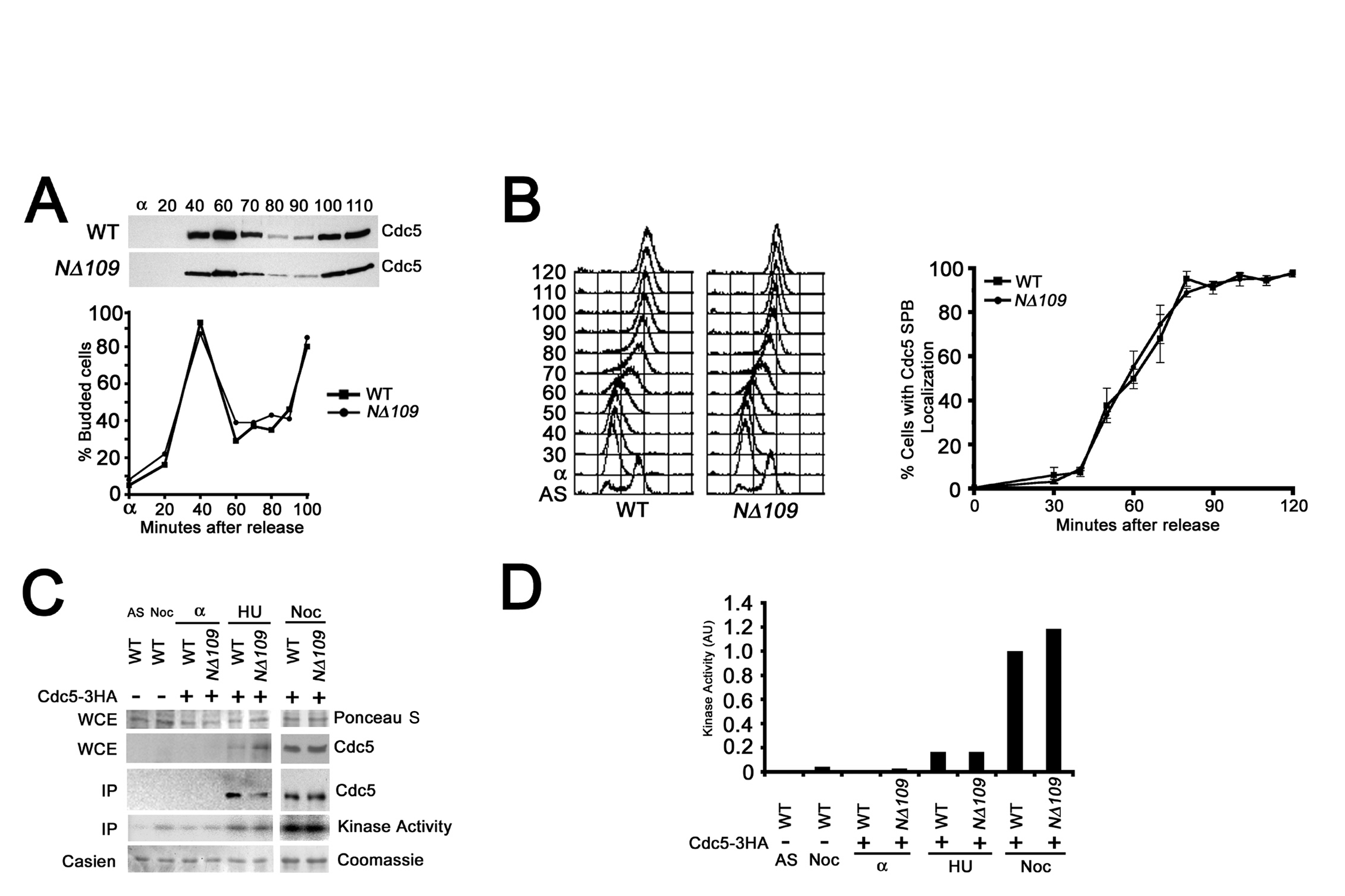

Supplement: Figure S2 — Cdc7p-Dbf4p does not alter Polo kinase abundance or activity. (A) WT (K6019) and dbf4-NΔ109 (M1874) containing CDC5-HA3 were arrested in G1, released into the cell cycle and blotted for Cdc5-HA3 protein. Budding index is shown. (B) DBF4 CDC5-4XGFP SPC42-eqRFP (M2750) and dbf4-NΔ109 CDC5-4XGFP SPC42-eqRFP (M2748) were arrested in G1 phase with mating pheromone and released into nocodazole. These were scored for Cdc5-4XGFP localization to the spindle pole body. (C) Strains expressing Cdc5-3HA protein were arrested at 30°C with alpha-factor (α) and released into YPD containing 0.2 M hydroxyurea (HU) or 15 µg/ml nocodazole (Noc) for 2 hours. Extracts were blotted for Cdc5 protein and Cdc5p kinase activity was measured following IP (D) Quantitation of kinase activity from three independent experiments. (0.75 MB TIF) [file pgen.1000498.s002.tif]

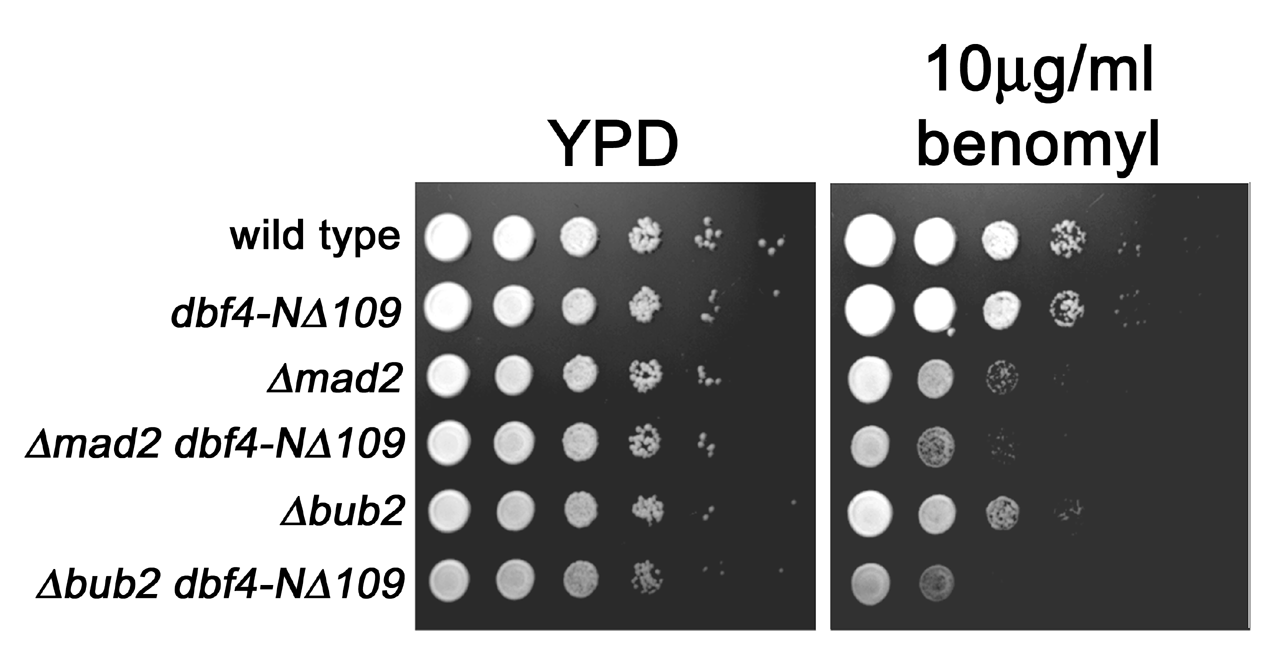

Supplement: Figure S3 — Deletion of the Dbf4p PIR causes decreased growth in the presence of spindle poisons. Indicated strains were spotted at 10-fold serial dilutions on YPD or YPD containing 10 µg/ml benomyl. (2.47 MB TIF) [file pgen.1000498.s003.tif]

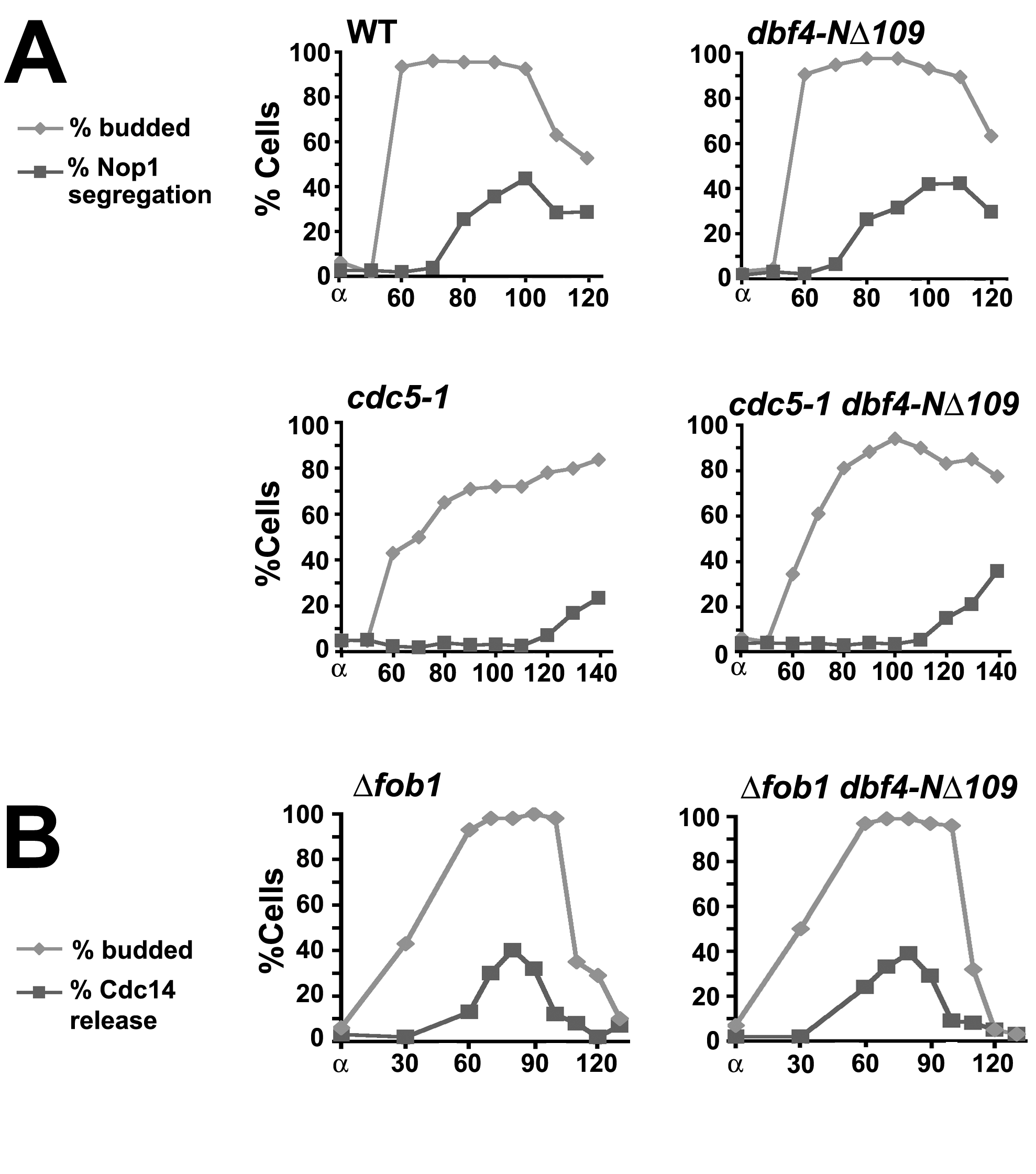

Supplement: Figure S4 — (A) Deletion of the Dbf4p PIR does not enhance nucleolar segregation in cdc5-1 cells. DBF4, dbf4-NΔ109, cdc5-1 and cdc5-1 dbf4-NΔ109 cells transformed with a centromeric EGFP-Nop1 plasmid were arrested in G1 with mating pheromone and released into the cell cycle in YPD at 34°C. Alpha-factor was added back after budding to permit a single cell cycle. Samples were taken at the indicated time points and scored for the presence of one or two GFP signals by florescence microscopy. (B) Deletion of the Dbf4p PIR does not enhance Cdc14 release when combined with deletion of FOB1. Δfob1 CDC14-EGFP (M3149) and Δfob1 dbf4-NΔ109 CDC14-EGFP (M3148) were arrested in G1 with mating pheromone and released into the cell cycle at 30°C. Alpha-factor was added back after budding to follow a single cell cycle. Samples were taken at the indicated time points and scored for release of Cdc14 from the nucleolus. (0.22 MB TIF) [file pgen.1000498.s004.tif]

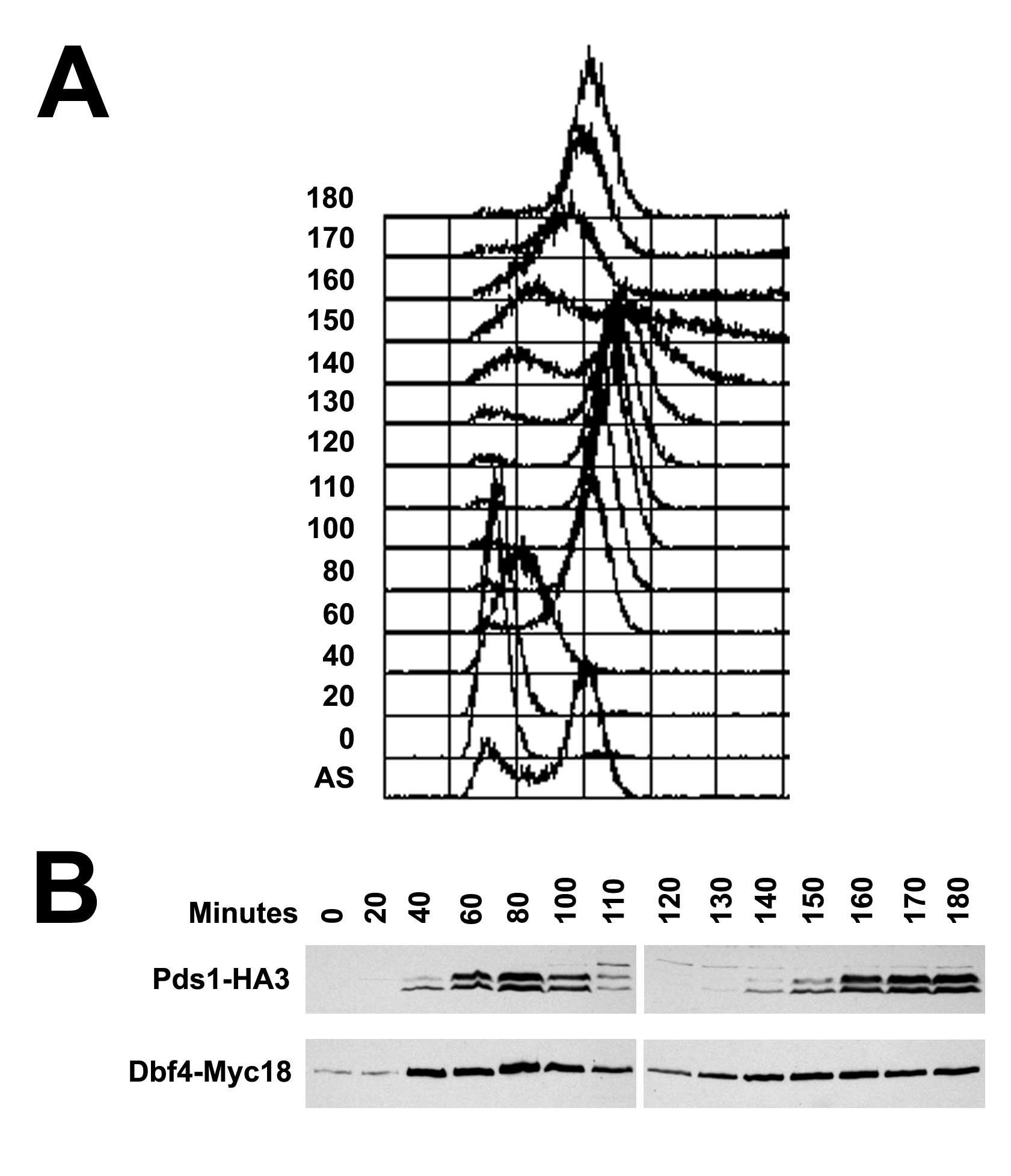

Supplement: Figure S5 — Some Dbf4p persists after Cdc20 activation during an unperturbed cell cycle. PDS1-HA3 DBF4-Myc18 (M3161) was arrested in G1 with mating pheromone and released into the cell cycle at 20°C. Samples were taken at the indicated time points. Protein extracts were made for Western blotting and cells processed for DNA content analysis by flow cytometry. Western blots were probed with 9E10 α-Myc (Dbf4-Myc18) and 12CA5 α-HA (Pds1-HA) antibodies. (3.24 MB TIF) [file pgen.1000498.s005.tif]
