# Supplementary material for: Cdc7p-Dbf4p Regulates Mitotic Exit by Inhibiting Polo Kinase
Source: PLoS Genet. 2009 May 29;5(5):e1000498. doi: 10.1371/journal.pgen.1000498 (PMC2682205; doi:10.1371/journal.pgen.1000498)
Supplement: Table S1 — Yeast strains used in this study. (0.08 MB DOC) [file pgen.1000498.s006.doc]

**Table S1. Yeast strains used in this s**tudy

| **Strain** | **Genotype** | **Source** |
| --- | --- | --- |
| W303-1A | *MAT***a** *ade2-1, ura3-1 his3-11, -15 trp1-1 leu2-3, -112 can1-100* | R. Rothstein |
| W303-1B | *MAT***α** *ade2-1, ura3-1 his3-11, -15 trp1-1 leu2-3, -112 can1-100* | R. Rothstein |
| K6019 | W303 *MAT***a** *CDC5-HA3-URA3* | K. Nasmyth |
| PJ69-4A | *MAT***a** *trp1-901 leu2-3, -112 ura3-52 his3-200 gal4∆ gal80∆ LYS2::GAL1-HIS3 GAL2-ADE2 met2::GAL7-lacZ* | E.A. Craig |
| RHC15 | W303 *MAT***a** *mad2∆::URA3* | A. Murray |
| M1261 | W303 *MAT***a** *dbf4-N∆109* | [26] |
| M319 | W303 *MAT***a** *dbf2-1* | [17] |
| M331 | W303 *MAT***a** *cdc15-2* | [17] |
| M358 | W303 *MAT***a** *mcm2-1* | [17] |
| M565 | W303 *MAT***a** *cdc14-1* | This study |
| M609 | W303 *MAT***a** *cdc14-3* | “ |
| M1614 | W303 *MAT***a** *cdc5-1* | “ |
| M1652  M1656 | W303 *MAT***a** *bub2∆::URA3*  W303 *MAT***a** *dbf4-N∆109-kanMX6* | “  “ |
| M1678 | W303 *MAT***a** *cdc5-2-URA3* | “ |
| M1804 | W303 *MAT***a** *dbf4-N∆109-kanMX6 cdc5-1* | “ |
| M1860 | W303 *MAT***a** *bub2∆::URA3 dbf4-N∆109-kanMX6* | “ |
| M1864 | W303 *MAT***a** *mad2∆::URA3 dbf4-N∆109-kanMX6* | “ |
| M1866 | W303 *MAT***a** *dbf4-N∆109-kanMX6* *cdc14-1* | “ |
| M1868 | W303 *MAT***a** *dbf4-N∆109-kanMX6 cdc14-3* | “ |
| M1870 | W303 *MAT***a** *dbf4-N∆109-kanMX6 dbf2-1* | “ |
| M1872 | W303 *MAT***a** *dbf4-N∆109-kanMX6 cdc15-2* | “ |
| M1874 | W303 *MAT***a** *CDC5-HA3-URA3* *dbf4-N∆109-kanMX6* | “ |
| M1918 | W303 *MAT***a** *kar9Δ::klTrp1* | “ |
| M1959 | W303 *MAT***α** *cdc5-2-URA3* *dbf4-N∆109-kanMX6* | “ |
| M1960 | W303 *MAT***a** *cdc5-2-URA3* *dbf4-N∆109-kanMX6* | “ |
| M1992 | W303 *MAT***a** *CDC14-EGFP-kanMX6* | “ |
| M2005 | W303 *MAT***a** *CDC14-EGFP-kanMX6* *dbf4-N∆109-kanMX6* | “ |
| M2007 | W303 *MAT***α** *dbf4-N∆65-kanMX6* | “ |
| M2139 | W303 *MAT***a** *CDC14-EGFP-kanMX6* *dbf4-N∆109-kanMX6 cdc5-1* | “ |
| M2179 | W303 *MAT***a** *kar9Δ::klTrp1 dbf4-N∆109-kanMX6* | “ |
| M2234 | W303 *MAT***a** *bub2Δ::ura3::HIS3* | “ |
| M2269 | W303 *MAT***a** *dyn1Δ::klTrp1* | “ |
| M2283 | W303 *MAT***a** *bub2∆::ura3::HIS3 dbf4-N∆109-kanMX6 cdc5-2-URA3* | “ |
| M2285 | W303 *MAT***α** *bub2∆::ura3::HIS3 dbf4-N∆109-kanMX6 cdc5-2-URA3* | “ |
| M2287 | W303 *MAT***a** *CDC14-EGFP-kanMX6* *cdc5-1* | “ |
| M2289 | W303 *MAT***a** *bub2∆::ura3::HIS3 cdc5-2-URA3* | “ |
| M2291 | W303 *MAT***α** *bub2∆::ura3::HIS3 cdc5-2-URA3* | “ |
| M2293 | W303 *MAT***a** *dyn1Δ::klTrp1 dbf4-N∆109-kanMX6* | “ |
| M2357 | W303 *MAT***a** *CDC5-3HA-URA3* *bar1∆::LEU2* | “ |
| M2359 | W303 *MAT***a** *CDC5-3HA-URA3* *dbf4-N∆109-kanMX6 bar1∆::LEU2* | “ |
| M2712 | W303 *MAT***a** *kar9Δ::klTrp1 dbf4-N∆109-kanMX6 cdc5-1* | “ |
| M2728 | W303 *MAT***a** *dyn1Δ::klTrp1 bub2∆::HIS3* | “ |
| M2741 | W303 *MAT***a** *CDC5-Myc15 3HA-CDC7* | “ |
| M2743 | W303 *MAT***a** *CDC5-Myc15 3HA-CDC7 dbf4-N∆109-kanMX6* | “ |
| M2748 | W303 *MAT***a** *CDC5-4xGFP-klTrp1 SPC42-eqFP-hphNT1* *dbf4-N∆109-kanMX6* | “ |
| M2750 | W303 *MAT***a** *CDC5-4xGFP-klTrp1 SPC42-eqFP-hphNT1* | “ |
| M2804 | W303 *MAT***a** *dbf4-N∆82-88-kanMX6* | “ |
| M2818 | W303 *MAT***a** /*MAT***α** *cdc5-1/cdc5-1* | “ |
| M2822 | W303 *MAT***a** /*MAT***α** *cdc5-1/cdc5-1 DBF4/dbf4-N∆109-kanMX6* | “ |
| M2826 | W303 *MAT***a** /*MAT***α** *cdc5-1/cdc5-1 dbf4-N∆109-kanMX6/dbf4-N∆109-kanMX6* | “ |
| M2908 | W303 *MAT***α** *cdc5-1 dbf4-N∆82-88-kanMX6* | “ |
| M3093 | W303 *MAT***a** [pMHY193; pRS316-GFP-Nop1] | “ |
| M3094 | W303 *MAT***a** *dbf4-N∆109-kanMX6* [pMHY193; pRS316-GFP-Nop1] | “ |
| M3095 | W303 *MAT***a** *cdc5-1* *dbf4-N∆109-kanMX6* [pMHY193; pRS316-GFP-Nop1] | “ |
| M3096 | W303 *MAT***a** *cdc5-1* [pMHY193; pRS316-GFP-Nop1] | “ |
| M3148 | W303 *MAT***a** *fob1Δ::his5* *dbf4-N∆109-kanMX6 CDC14-EGFP-kanMX6* | “ |
| M3149 | W303 *MAT***a** *fob1Δ::his5 CDC14-EGFP-kanMX6* | “ |
| M3161 | W303 *MAT***a** *DBF4-Myc18-LEU2 PDS1-HA3-LEU2* | “ |
