# Supplementary material for: Cdc7p-Dbf4p Regulates Mitotic Exit by Inhibiting Polo Kinase
Source: PLoS Genet. 2009 May 29;5(5):e1000498. doi: 10.1371/journal.pgen.1000498 (PMC2682205; doi:10.1371/journal.pgen.1000498)
Supplement: Table S2 — Plasmids used in this study. (0.05 MB DOC) [file pgen.1000498.s007.doc]

**Table S2. Plasmids used in this study**

| **Plasmid** | **Description** | **Source** |
| --- | --- | --- |
| pMHY193 | pRS316-GFP-Nop1 | M. Henry |
| pCG10 | pRS415-*DBF4_110-704_* | [26] |
| pCG53 | pGBKT7-*DBF4_67-704_* | This study |
| pCG60 | pGBKT7-*DBF4_67-_*_227_ , *ADH1* promoter Δ | “ |
| pCG61 | pCG60-*DBF4_110-227_* | “ |
| pCG74 | pGBKT7-*DBF4_110-704_* | “ |
| pCG162 | pRS416-*pGAL1, 10* | “ |
| pCG163 | pCG162*-**DBF4_109-225_* | “ |
| pCG166 | pCG162*-DBF4_1-225_* | “ |
| pCG167 | pCG162*-DBF4_65-225_* | “ |
| pCG213 | *pGAL-DBF4_1-225, Δ 82-88_* | “ |
| pCM3 | pGAD-*CDC5_454-705_* | “ |
| pCM4 | pGAD-*CDC5_510-705_* | “ |
| pCM16 | pAcSG2-*3Myc-CDC5_65-705_* | “ |
| pCM21 | pCG60-*DBF4_67-109_* | “ |
| pCM24 | pCM21, S84A, S92A, T95A, T105A | “ |
| pGAD-CDC5.3 | pGAD-*CDC5_422-705_* | “ |
| pHS4 | pSUMO-*DBF4_66-109_* | “ |
| pMW489 | pRS415-*DBF4* | [26] |
| pMW535 | pRS415-*CDC5* | “ |
| pMW537 | pGEX-KG-*CDC5_357-705_* | “ |
| pYJ38 | pCG60-*DBF4_67-227 Δ82-88_* | “ |
| pYJ150 | pRS425-*DBF4_110-704_* | “ |
| pYJ152 | pRS425-*DBF4**_Δ82-88_* | “ |
| pYJ154 | pRS425-*DBF4* | “ |
| pYJ161 | pRS425-*DBF4_ΔC312-704_* | “ |
| pYJ162 | pRS425-*DBF4_Δ65 + ΔC312-704_* | “ |
| pYJ163 | pRS425-*DBF4_Δ109 + ΔC312-704_* | “ |
| pYJ164 | pRS425-*DBF4_Δ82-88 + ΔC312-704_* | “ |
| pYJ204 | pGBKT7-*DBF4 _1-704_* | “ |
| pYJ206 | pGBKT7-*DBF4 _1-704, Δ82-88_* | “ |
