# Supplementary material for: Cdc7p-Dbf4p Regulates Mitotic Exit by Inhibiting Polo Kinase
Source: PLoS Genet. 2009 May 29;5(5):e1000498. doi: 10.1371/journal.pgen.1000498 (PMC2682205; doi:10.1371/journal.pgen.1000498)
Supplement: Text S1 — Supplementary methods: Cdc5 protein abundance, kinase activity, and SPB localization; nucleolar segregation assay; and Dbf4 cell cycle abundance. (0.03 MB DOC) [file pgen.1000498.s008.doc]

**Supplementary Methods:**

**Cdc5 Protein Abundance, Kinase Activity and SPB Localization**

To analyze Cdc5 protein abundance during the cell cycle K6019 and M1874 were arrested with alpha-factor (5μg/ml) for 3h, released into the cell cycle at 30^o^C in YPD and TCA extracts were analyzed by Western blotting. Cdc5-HA3p kinase activity was analyzed in asynchronous, G1 (0.1μg/ml alpha-factor), HU (0.2M) and nocodazole (15μg/ml) arrested cultures. Bead-beated whole cell extracts from strains M2357 and M2359 were made in (20mM Tris-HCl, pH7.4, 150mM NaCl, 0.5% NP-40, 1mM EGTA), immunoprecipitated with 12CA5-protein A Sepharose and washed 4x in the same buffer. Kinase activity was measured on half of the IP using casein as a substrate in (50mM Tris-HCl, pH 7.5, 10mM McCl_2_, 5mM DTT, 2mM EGTA, 100μM ATP, 10μCi [γ-^32^P] ATP). Bound proteins were separated on 10% SDS-PAGE and visualized by autoradiography or by Western blotting. To examine Cdc5p SPB localization, G1 arrested cells (M2748, M2750) were released into the cell cycle at 20°C in YPD media containing 15μg/ml nocodazole. Cells were fixed in 4% paraformaldeyde for 10 minutes, stained with DAPI (1μg/ml) and analyzed for Cdc5-4xGFP spindle pole body localization using fluorescence microscopy.

**Nucleolar Segregation Assay**

To assess nucleolar segregation *DBF4*, *dbf4-NΔ109, cdc5-1* and *cdc5-1 dbf4-NΔ109* cells transformed with a centromeric EGFP-Nop1 plasmid (pMHY193) were arrested in G1 with mating pheromone and released into the cell cycle in YPD at 34°C. Alpha-factor was added back after budding to permit a single cell cycle. Samples were taken at the indicated time points and scored for the presence of one or two GFP signals by florescence microscopy.

**Dbf4 Cell Cycle Abundance**

M3161 expressing Pds1-HA3p and Dbf4-Myc18p was arrested in G1 at 25°C with mating pheromone and released into the cell cycle at 20°C. Samples were taken at the indicated time points. Protein extracts were made for Western blotting by the TCA method [71] and cells were processed for DNA content analysis by flow cytometry [17]. Western blots were probed using 9E10 (1:1000) and 12CA5 (1:1000) antibodies to detect Dbf4-Myc18p and Pds1-HA3p, respectively.
